# Optimization of Performance and Emission Characteristics of Catalytic Coated IC Engine with Biodiesel Using Grey-Taguchi Method

**DOI:** 10.1038/s41598-019-57129-9

**Published:** 2020-02-07

**Authors:** Sureshbabu Yessian, P. Ashoka Varthanan

**Affiliations:** 1 Department of Mechanical Engineering, Sri Eshwar College of Engineering, Coimbatore, Tamilnadu India; 2 Department of Mechanical Engineering,Sri Krishna College of Engineering & Technology, Coimbatore, Tamilnadu India

**Keywords:** Climate sciences, Environmental sciences, Environmental social sciences, Chemistry, Energy science and technology, Materials science

## Abstract

The performance and emission affected by piston catalytic coating have been analyzed in this study. The main contributing factors for improving performance such as load, fuel and speed have been preferred to reduce the emission and improve the performance of an IC engine. These specific standard parameters have been modified with copper alloy coated diesel engine with the aid of design of experiment by Taguchi with grey relational analysis optimization (GRA) method, for improving the IC engine performance and reducing the emissions. The result shows modified copper chromium zirconium (CuCr1Zr) catalytic coated piston produces less emission and improves performance when compared to standard un-coated piston type engine. In this investigation cotton seed oil is used as a bio- diesel and the piston and combustion chamber were coated with copper chromium zirconium material with a thickness of 250 microns. Finally the results of the experiments were compared with un-coated engine and optimized parameters have been identified for catalytic coated modified IC engine using Taguchi with GRA approach.

## Introduction

IC engine is the main power source of automotive vehicles. The engine consists of many parts such as piston, cylinder head, cylinder block, etc. Piston and cylinder head are the most important components of IC engine because the power of IC engine depends upon the combustion taking place in combustion chamber. It is located above the piston and bottom of the cylinder head. As the result of the combustion pollutants are produced. Combustion is burning of fuel combined with air. In this chemical process, fuel like hydro carbon combined with oxygen and produces harmful gases leading to air pollution. During the combustion process, oxidization, does not occur properly. So, carbon monoxide is produced, and hydro carbon emission occur due to light load as the lean mixture^1^, So flame speed during the combustion may be too low or in-complete combustion may be occur^2^ and hence emission level of HC, CO and NOx in the exhaust gas discharged into the atmosphere through tail pipe is higher compared to copper coated piston type engine. These gases cause many drawbacks to the society. To overcome this problem, catalytic material (copper -chromium -zirconium) is used in the form of 250 -micron coating over the piston crown and combustion chamber walls. The coating of piston and combustion chamber is done using plasma spraying process of thermal spraying technique. Rameshbabu *et al*. proved that the catalytic coating, reduced the required ignition energy and the flame velocity is increased^3^. Winkler M. F *et al*. stated that coated diesel engine gives better performance^4^. Jeyakumar *et al*., state that cotton seed bio diesel can be used as an alternative fuel for control the emissions like CO, HC of a diesel engine^5^.

## Materials and Method

**Figure 1:**
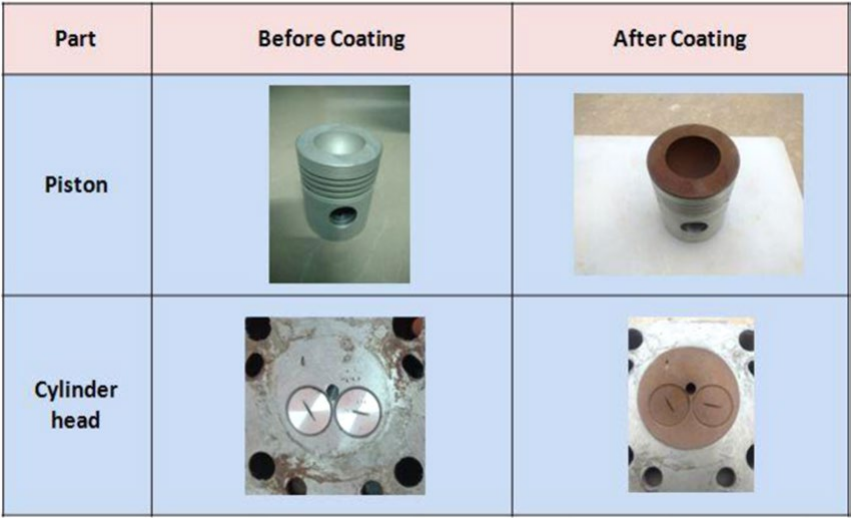
Piston, and Combustion chamber coated by plasma coating technique- The figure describe before and after coating (Own Image -Photograph taken by corresponding author at the time of research work at Coimbatore).

The experimental works were conducted in catalytic coated IC engine. The engine was tested with diesel, cotton seed oil blending with diesel fuel with the ratio of 10% to 20% and by varying the engine performance and emissions parameters like load and speed. The experiments are conducted based on taguchi orthogonal array (OA). A Minitab ’16 statistical software is used for selection of OA. Based on OA, the design of experiments (DOE) are made. The experiments are conducted in 150cc kirlosker make multi fueled operated diesel engine with eddy current dynamometer and gas analyzer set up. The setup enables to find the coated engine emission characteristics like carbon monoxide (CO), hydro carbon (HC) and oxides of nitrogen (NOx) and the important coated engine performance like brake power (BP), brake thermal efficiency Bth and torque. Patil. K. R *et al*. investigated the diesel engine emission and conducted the performance test as per ISO 8178-C1 and ISO 8178-D2 procedure^6^. CO is measured using Non dispersive infrared (NDIR) sensor, HC using Flame ionization detector (FID) and NOx using chemiluminescent analyzer. The main aim of the paper is to minimize the major diesel engine emissions like CO and HC. In this research, A DC-5 GAS analyzer is used for measuring the emission levels. The measuring probe of DC- GAS analyzer is connected to engine exhaust tailpipe or muffler. It measures the five emission gases, including hydrocarbons (HC), carbon monoxide (CO), and oxides of nitrogen (NOx) in the exhaust gas. Ponnusamy^7^ *et al*. investigated the performance evaluation of single cylinder IC engine. The results showed that copper coated piston and combustion chamber engine reduces HC and CO emissions. He also carried out the investigation for different catalytic coating materials. Finally they rated catalysts based on the performance as copper > chromium > nickel > standard or un-coated (aluminum alloy). Krzysztof *et al*. Studied the effects of plasma sprayed zirconium coatings on the piston^8^. So, one of the promising technologies for improving IC engine performance and reducing the CO and HC emission is catalytic coating on piston and combustion chamber. Since copper chromium and zirconium are less in cost compared to platinum, and also they are is a good catalyst material, these three materials combined in the form of alloys material (CuCr1Zr), and used for coating. So in this investigation, copper -chromium -zirconium material was selected as the coating material is shown in Fig. 1 It is most suitable for internal combustion engines and also good corrosion resistance.

### Methodology

**Figure 2:**
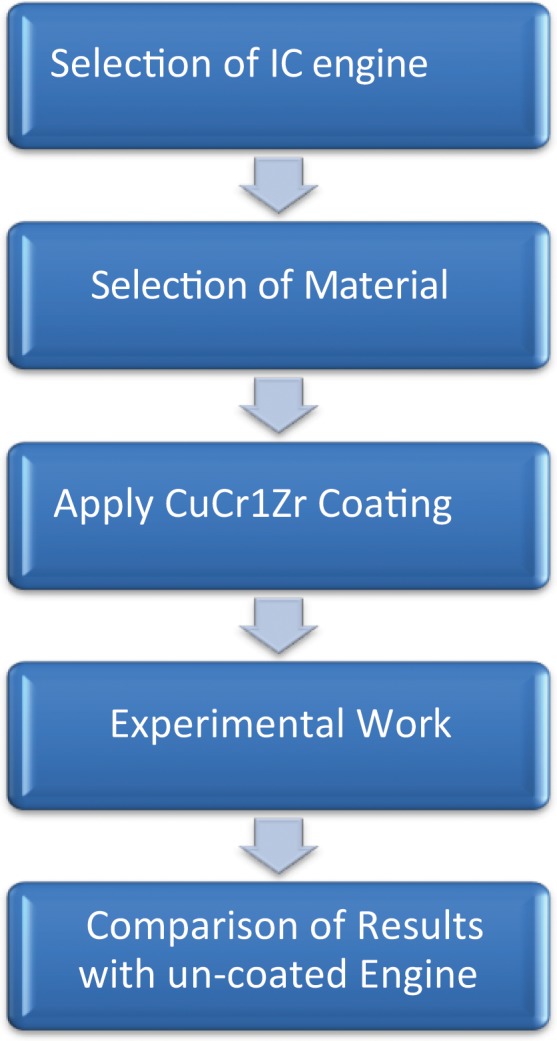
Methodology -The flowchart indicates step-by step process involved in the present investigation.

To find the catalytic coated engine performance and emission parameters the following procedure is followed as shown in Fig. 2.

### Plasma coating

**Figure 3:**
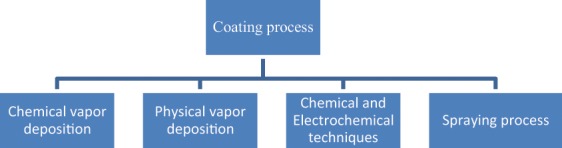
Classification of Coating Process - The figure shown different type of thermal spray coating process.

The plasma spray method is basically a thermal spraying coating process. The material to be coated is converted into molten stage by means of heat and sprayed to the surface to be coated and produce a coating. It is shown in Fig. 3

**Figure 4:**
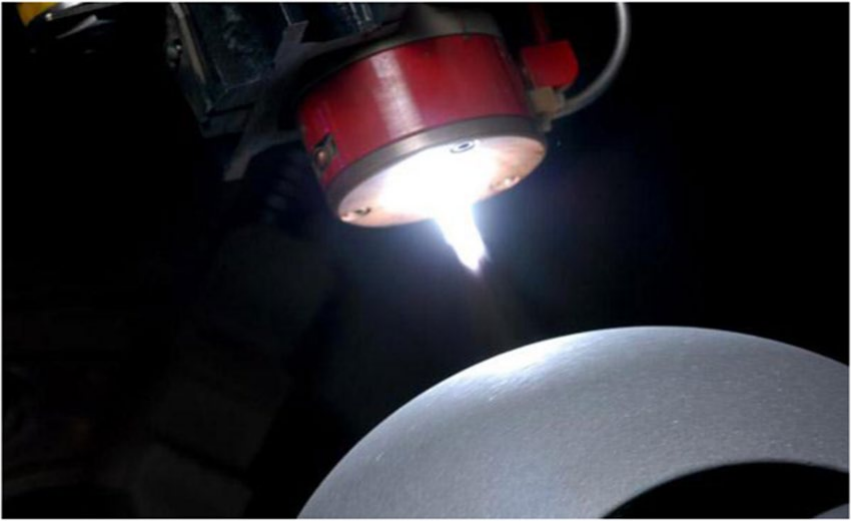
Plasma Coating Process-The figure describe the Piston and head coated by CUCr1Zr material (Own Image -Photograph taken by corresponding author at the time of piston coating at Bangalore).

The coating material impacts on the substrate surface and quickly cools forming a coating^9^. The plasma coating method has been shown in Fig. 4. below.

### Piston coating

Today‘s engines uses an exhaust after gas treatment system which is otherwise called as catalytic converter. This investigation intends to implement a similar kind of technology used in catalytic converter in the engine itself. This is archieved by coating the piston and chamber thereby making it possible to reduce a major amount of pollution emitted by the engine. So, this investigation involves coating of piston and combustion chamber with a catalytic material. Copper-chromium-zirconium alloy is coated on the bottom side of engine cylinder head and the top side of piston crown to a thickness of 250 microns. There are different methods of catalytic coating process for coating the piston and combustion chamber walls as shown in Fig. 3. The plasma spray process is advised by many researchers^9–11^ because of its performance, economy and eco friendly nature. The properties of coating material values are tabulated in Table 1

**Table 1:** Properties of copper chromium zirconium material.

S. No	Parameters	Values
1	Tensile Strength	220–540 N/mm^2^
2	0.2% Proof Strength	100–440 N/mm^2^
3	Elongation	5–35%
4	Hardness (HV)	55–175
5	Electrical Conductivity	80% IAC
6	Thermal Conductivity	300 W/m°K
7	Melting Point	1080 °C

The surface roughness of the coating was measured using Mitutyo make Surf test SJ-210 testing machine. The average surface roughness value before coating is Ra = 6.2 ± 0.3 μm and after coating is Ra = 5.9 ± 0.4 μm. Hence the surface roughness after coating has improved.

Kadir Mert Doleker *et al*. was conducted porosity measurements of thermal barrier coatings. The results showed that that the thermal barrier coatings exhibit higher porosity^12^. Das. D *et al*. Conducted investigation on partially stabilized zirconium coated piston with the thickness of 250, 350 and 450 microns. The results showed that improvement of brake thermal efficiency and reduction of SFC, CO, HC emissions^13^. In this investigation the piston is coated with CuCr1Zr to a thickness of 250 microns and it does not affect the compression ratio.

## Experimental Setup

**Figure 5:**
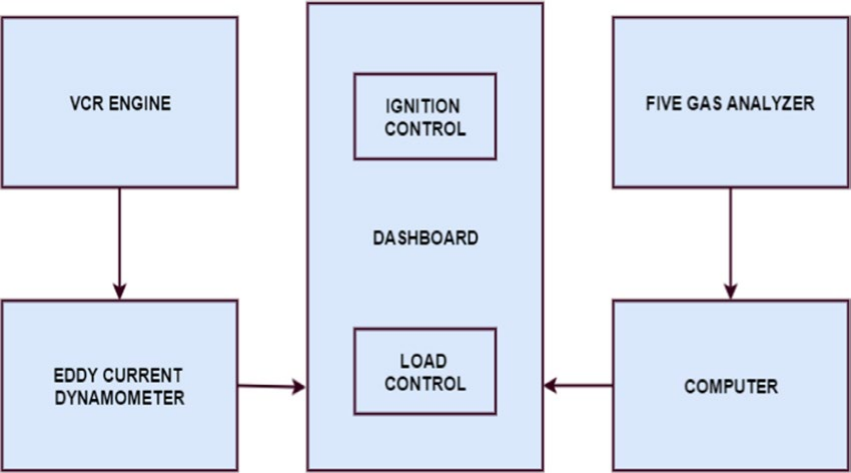
Experimental set up Various Components -The figure shown the connections of each element.

**Figure 6:**
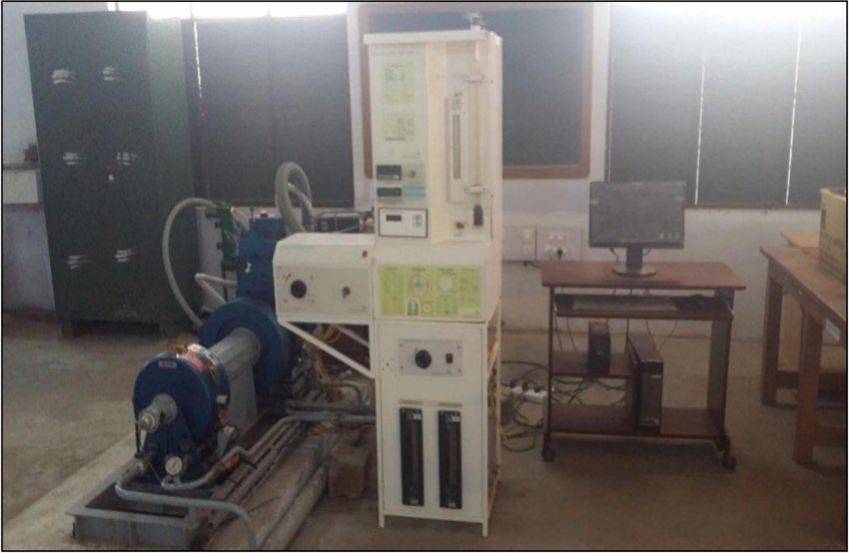
Experimental setup –The Engine Connected with Eddy Current Dynamometer and Exhaust Gas Analyzer (Own Image -Photograph taken by corresponding author during experiment conducted).

**Figure 7:**
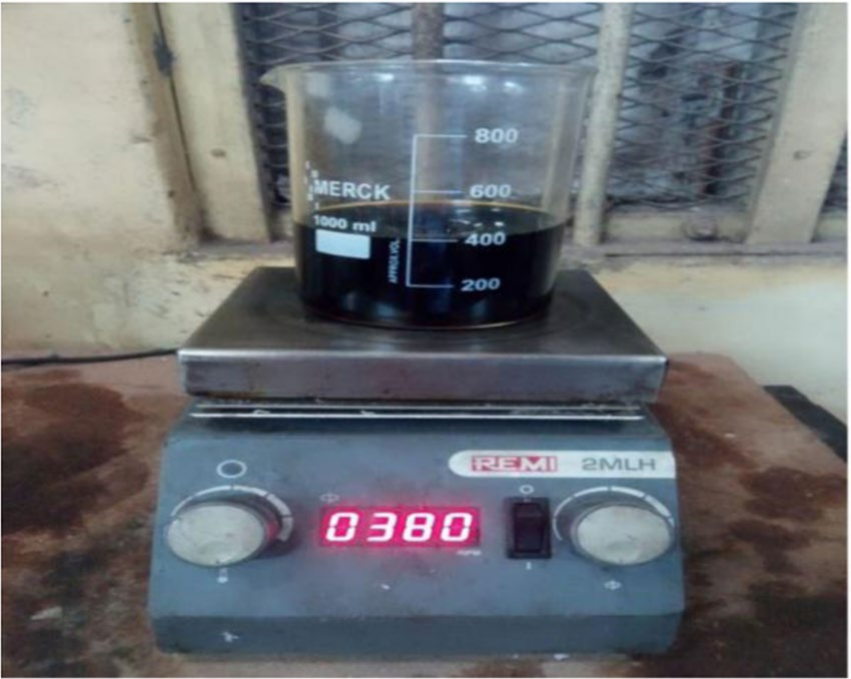
Blending of Cotton Seed bio oil mixing with Diesel Fuel – The figure shown cotton seed bio oil stirring with diesel fuel and heating. (Own Image -Photograph taken by corresponding author at the time of research work).

The experimental set up consists of five different elements. The five various elements are catalytic coated engine, eddy current dynamometer, gas analyzer, personnel computer and data acquisition system. These elements are connected as shown in Figs. 5 and 6. This set up is used for conducting the engine performance test and emission test. The engine can be operated by two fuels either by petrol or diesel. If the engine is operating in petrol fuel, the electronic control unit (ECU) is needed. In this research, the engine is run by diesel fuel. Here, it operated with the help of injector and fuel pump (FIP). The experiments are conducted using diesel and blended diesel 10% and 20%, as shown in Fig. 7.

### Bio diesel preparation

In this investigation cotton seed oil is used as blended diesel fuel. Cotton seed oil is produced from the seed of the cotton plant, by crushing or by chemical solvent extraction process. For commercial purpose, cottonseed oil is extracted through solvent extraction process. The properties of cotton seed oil is listed in Table 2.

**Table 2:** Properties of cotton seed oil.

S. No	Parameters	Values
1	Density	857 Kg/m^3^
2	Flash point	198 °C
3	Fire point	225 °C
4	Calorific value	35 MJ/Kg
5	Cetane number	38

**Figure 8:**
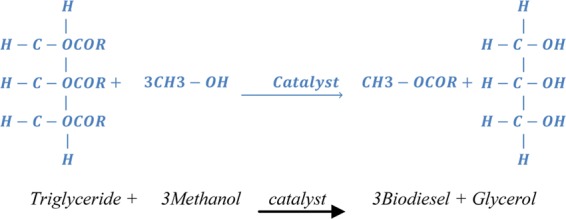
Transesterification Chemical Reaction-The figure describe the converting reaction of cotton seed oil into biodiesel by adding of methanol and one ester group converted into another ester group and glycerol.

Cotton seed plant (Gossypium hirsutum and Gossypium herbaceum) is grown by farmers for the purpose of feeding animals, make cotton cloths, and produce cotton oil. The seed of cotton has a similar shape to sunflower seed. Both seeds contains oil contaminants inside the hull. Using the chemical extraction process, the cotton seed oil is extracted from the kernel. Now-a-days due to high demand and shortages of crude oil, the cost of automotive vehicle fuel increases day by day^14,15^. So the cottonseed oil can be used as an alternative fuel for automotive vehicles. In this investigation cotton seed oil is blending with diesel fuel with the ratio of 10% to 20% (B10, B20) using transesterification process. In the transesterification process one ester group is converted or interchanged into another ester group. The converting reaction of cotton seed oil into biodiesel is called transesterification process is shown in Fig. 8. In this process, methanol and alcohol combine with the triglyceride oil in the cotton seed under heat and sodium or potassium hydroxide as catalyst. Bio diesel and glycerol is produced through the chemical reaction. Ayegba, *et al*.^14^ describe, the transesterification reaction as shown in Fig. 8. After the sedimentation process, glycerol and bio diesel is separated. The properties of cotton seed oil is compared with diesel fuel in Table 3.

**Table 3:** Comparision of Diesel and Biodiesel fuel properties.

S. No	Parameters	Diesel	Cottonseed oil
1	Density (kg/m^3^)	830 kg/m^3^	857 kg/m^3^
2	Flash point (°C)	65 °C	198 °C
3	Fire point (°C)	107 °C	225 °C
4	Calorific value (MJ/Kg)	42.0 MJ/Kg	35.86 MJ/Kg
5	Cetane number	40–55	38

### Taguchi method

Taguchi with grey relational analysis (GRA) is most suitable technique for multi-performance characteristics with minimum experimental work^16^. In the present investigation, taguchi technique with grey rational analysis method is used for finding the optimum engine parameters for coated IC engine. To find the optimum solution of a problem with minimum number of trails, taguchi technique is the most preferable method. This technique uses an orthogonal array concept. In the present study, three factors and three levels L9 orthogonal array is used. Taguchi`s array selector table used for selecting orthogonal array^17^. The identified parameter should be in three levels such as smallest, medium and highest levels. Taguchi technique uses a concept known as signal to noise quantitative relation (S/N) for measuring the standard characteristics.

### Grey rational analysis (GRA) method

Goutam Pohit *et al*. used the grey rational analysis for multi objective problems^18^. The intention of the investigation is to optimize seven response parameters for coated piston and head type IC engine. Out of seven responses, four responses related to engine performance and remaining three responses related to emission. In this, higher S/N ratio is preferred for engine performance and lower S/N ratio for emission characteristic. Therefore, taguchi is not suitable for multi response optimization problem. To overcome this problem, in this investigation taguchi with grey relational analysis is used to find the optimum solution.

Prasanta Sahoo *et al*. used grey rational technique^19^. The first step is experimental results are normalized as the first step. After normalization, the grey relational coefficients (GRC) are calculated in the second step. In the third step, the overall grey relational grade (GRG) is calculated for each selected response by averaging the grey relational coefficients. Finally, evaluation of the multiple process response is based on the grey relational grade.

### Parameters and its levels

In the present investigation three factors and three levels are used. Experiments are conducted considering three input parameters such as Fuel, Load (%) and Speed (rpm). Overall nine experiments are carried out. Table 4 shows the values of various parameters used for experiments.

**Table 4:** Factors and its levels.

S. No	Factors	Level 1	Level 2	Level 3
1	Fuel	Diesel	B10	B20
2	Load (%)	0	50	100
3	**Speed** (rpm)	1460	1480	1500

Minitab-16 statistical software is used in this investigation. The parameter variation levels are updated in Minitab-16 statistical software, and the software suggests that L9 (3*3) orthogonal array as shown in Table 5. The experiments are performed for the diesel and blended bio diesel with the proportion of 10% and 20% bio diesel with 90% and 80% diesel fuel (B10, B20).

**Table 5:** L9 Orthogonal array Design of Experiment.

Exp. No	Fuel Type	Load (%)	Speed (rpm)
1	Diesel	0	1460
2	Diesel	50	1480
3	Diesel	100	1500
4	B10	0	1480
5	B10	50	1500
6	B10	100	1460
7	B20	0	1500
8	B20	50	1460
9	B20	100	1480

## Result and Discussion

The experiments were conducted for the diesel and biodiesel B10, B20 on a coated piston engine and Table 6 shows engine performance results obtained through the experiments.

**Table 6:** Performance and Emission Results.

Exp.No	Fuel	Load (%)	Speed (rpm)	BP (kw)	SFC (kg/kw-hr)	BTh (%)	Torque (N-m)	CO (%)	HC ppm	Nox ppm
1	Diesel	0	1460	0.17	2.40	3.48	1.05	0.05	0.17	98
2	Diesel	50	1480	1.74	0.37	22.54	11.19	0.04	12	306
3	Diesel	100	1500	3.43	0.26	32.03	22.60	0.02	7	332
4	B10	0	1480	0.14	2.18	4.04	0.87	0.07	36	89
5	B10	50	1500	1.74	0.32	27.96	11.20	0.01	8	182
6	B10	100	1460	3.13	0.26	34.51	20.53	0.01	1	212
7	B20	0	1500	0.16	1.91	5.13	1.00	0.05	20	55
8	B20	50	1460	1.67	0.33	29.80	10.76	0.02	9	173
9	B20	100	1480	3.42	0.26	37.20	22.52	0.01	7	225

### GRA for performance and emission

The first step of GRA is normalizing the responses. The important engine performance responses are BP, brake thermal efficiency (BTh), and Torque. These are the important responses for coated engine. When the required response is higher the better, then the original sequence is normalized as per the Eq. (1)^20^.

1 $$\begin{array}{rcl}xi(k) & = & \frac{yi(k)-\,{\rm{\min }}\,yi(k)}{{\rm{\max }}\,yi(k)-\,{\rm{\min }}\,yi(k)}\\ xi(k) & = & \frac{0.17-0.14}{3.42\,-\,0.14}\\ xi(k) & = & 0.009\end{array}$$

The SFC, CO, HC and NO_X_ are also important emission responses of coated engine. Rajesh^21^ *et al*. used Smaller the Better, option to normalized parameters. The parameters are normalized as per the Eq. (2).

2 $$\begin{array}{rcl}xi(k) & = & \frac{{\rm{\max }}\,yi(k)-yi(k)}{{\rm{\max }}\,yi(k)-\,{\rm{\min }}\,yi(k)}\\ xi(k) & = & \frac{0.07-0.05}{0.07-0.01}\\ xi(k) & = & 0.333\end{array}$$

Similarly the remaining calculations are made as mentioned above, and it is tabulated in Table 6. (Where i = 1, 2, 3…, 9 experiments and k = 1, 2, 3, 4, 5, 6 and 7 for BP, SFC, BTh, Torque, CO, HC and NOX). The GRA performance and emission results are shown in Table 7

**Table 7:** GRA Performance and Emission Results.

Fuel	Load (%)	Speed (rpm)	BP (KW)	SFC (Kg/KW-hr)	BTE (%)	TORQUE (N-m)	CO (% vol.)	HC (ppm)	NOX (ppm)
Diesel	0	1460	0.09	0.00	0	0.08	0.33	1	0.84
Diesel	50	1480	0.48	0.94	0.5	0.47	0.5	0.66	0.09
Diesel	100	1500	1	1.00	0.8	1	0.83	0.80	0
B10	0	1480	0	0.10	0.01	0	0	0	0.87
B10	50	1500	0.48	0.97	0.72	0.47	1	0.78	0.54
B10	100	1460	0.90	1.00	0.92	0.90	1	0.97	0.43
B20	0	1500	0.01	0.22	0.04	0.005	0.33	0.44	1
B20	50	1460	0.46	0.96	0.78	0.45	0.83	0.75	0.57
B20	100	1480	0.99	1.00	1	0.99	1	0.80	0.38

### GRC and GRG for performance and emission

The grey relational coefficient (GRC) $\xi i(k)$ was obtained using Eq. (3). Where, *φ* is taken as 0.5 for equal preference. It is defined as identification coefficient^22^.

3 $$\xi i(k)=\frac{\Delta \,\min \,+\varphi \Delta \,\max }{\Delta 0ik+\varphi \Delta \,\max }$$

4 $$\Delta 0i=||x0(k)-x(k)||$$

The grey relational coefficient $\xi i(k)$ and Grey relational grade (*γi*) for the BP of the first experiment. Using the above equation is given below

$$\Delta 0i=1-0.0091=0.991$$

Where, ∆*min* = 0; ∆*max* = 1

$$\xi (k)=\frac{0+0.5(1)}{0.990\,+\,0.5(1)}$$

$$\xi i(k)=0.3355$$

Similarly the remaining calculations were made.

After finding the grey rational coefficient (GRC) the average grey rational coefficient (GRG) is calculated from each response. Based on the following equation, the overall grey relational grade (GRG) is calculated and it is tabulated in Table 8

5 $$\begin{array}{rcl}\gamma i & = & 1/n\,\varSigma \,\xi i({\rm{k}})\\ \gamma i & = & 1/7(\xi i(1)+\xi i(2)+\xi i(3)+\xi i(4)+\xi i(5)+\xi i(6)+\xi i(7))\\ \gamma i & = & 1/7(0.335+0.33+0.33+0.352+0.427+1+0.757)\\ \gamma i & = & 0.5044\end{array}$$

**Table 8:** GRG Performance and Emission Results.

Fuel	Load (%)	Speed (rpm)	BP (KW)	SFC (Kg/KW-hr)	BTE (%)	TORQUE (N-m)	CO (%vol.)	HC (ppm)	NOX (ppm)	GRG
Diesel	0	1460	0.336	0.333	0.333	0.335	0.428	1.000	0.763	0.509
Diesel	50	1480	0.493	0.900	0.534	0.484	0.500	0.602	0.355	0.556
Diesel	100	1500	1.000	1.000	0.764	1.000	0.749	0.723	0.333	0.799
B10	0	1480	0.333	0.350	0.337	0.333	0.333	0.333	0.802	0.402
B10	50	1500	0.493	0.940	0.645	0.489	1.000	0.695	0.521	0.686
B10	100	1460	0.845	1.000	0.862	0.839	1.000	0.955	0.468	0.860
B20	0	1500	0.334	0.390	0.344	0.334	0.428	0.474	1.000	0.473
B20	50	1460	0.483	0.938	0.694	0.478	0.749	0.669	0.539	0.653
B20	100	1480	0.994	1.000	1.000	0.992	1.000	0.723	0.448	0.885

The obtained grey relational rank is presented in Table 9 where higher grey relational grade is ranked high. The obtained higher grey rational grade is very closer to the optimum solution.

**Table 9:** Performance and Emission Results GRG Rank List.

Fuel	Load (%)	Speed (rpm)	BP (KW)	SFC (Kg/KW-hr)	BTE (%)	TORQUE (N-m)	CO (%vol.)	HC (ppm)	NOX (ppm)	GRG	RANK
Diesel	0	1460	0.336	0.333	0.333	0.335	0.428	1.000	0.763	0.509	7
Diesel	50	1480	0.493	0.900	0.534	0.484	0.500	0.602	0.355	0.556	6
Diesel	100	1500	1.000	1.000	0.764	1.000	0.749	0.723	0.333	0.799	3
B10	0	1480	0.333	0.350	0.337	0.333	0.333	0.333	0.802	0.402	9
B10	50	1500	0.493	0.940	0.645	0.489	1.000	0.695	0.521	0.686	4
B10	100	1460	0.845	1.000	0.862	0.839	1.000	0.955	0.468	0.860	2
B20	0	1500	0.334	0.390	0.344	0.334	0.428	0.474	1.000	0.473	8
B20	50	1460	0.483	0.938	0.694	0.478	0.749	0.669	0.539	0.653	5
B20	100	1480	0.994	1.000	1.000	0.992	1.000	0.723	0.448	0.885	1

Since the experimental plan is orthogonal the results of every parameter of grey relational grade are separated in to level. For fuel parameter, the mean of the gray relative grade for the various levels one, two and three are calculated by averaging the gray relational grade (GRG) for the experiments one to three, four to six and seven to nine. Similarly, the GRG for the remaining parameters load and speed are calculated as shown in Table 10.

**Table 10:** GRG Performance and Emission Results.

Symbol	Parameters	Level1	Level 2	Level 3	Main effect (max-min)	Rank
A	Fuel	0.621	0.649	0.670	0.0213	3
B	Load	0.461	0.632	0.848	0.2163	1
C	Speed	0.674	0.614	0.653	0.0215	2

Based on grey prediction concept, the higher grey rational grade input parameter (A3B3C1) is optimum. Therefore B20 fuel, 100% load and 1460 rpm engine speed is the optimum parameter for catalytic coated piston type diesel engine.

### ANOVA analysis

Raggul^23^ *et al*. stated that the main intension of ANOVA analysis is to identify and investigate the significant factor contribute to the engine emission and performance characteristics of a catalytic coated IC engine. The ANOVA analysis is carried out through the sum of squared deviations of the total mean of the GRG. Based on contribution and error of each factor, the effect of each experimental factor can be separated. The factor that poses the maximum mean square value is identified as the most significant parameter and the factor influences the performance and emission characteristics of a catalytic coated IC engine. The result of ANOVA is shown in Table 11. Patel^24^ *et al*. where stated that the sum of squared error (without or with pooled factor), is the sum of squares corresponding to the insignificant factors. (MSj) is Mean square of a factor found by dividing its sum of squares and degrees of freedom, and (ρ) is the percentage contribution of each of the design parameters. Degree of freedom for each factor is 2 (Number of level-1).

**Table 11:** ANOVA of GRG.

Factor	Degrees of freedom	Sum of Squares	Mean Squares	F ratio	Percentage Contribution (*ρ*)
Fuel (A)	2	0.0011	0.0006	0.11	0.777
Load (B)	2	0.0881	0.0440	6.98	0.045
Speed (C)	2	0.0029	0.0014	0.48	0.178
Error	2	0.0041	0.0020	—	0
Total	8	0.9631	0.0480	—	100

**Figure 9:**
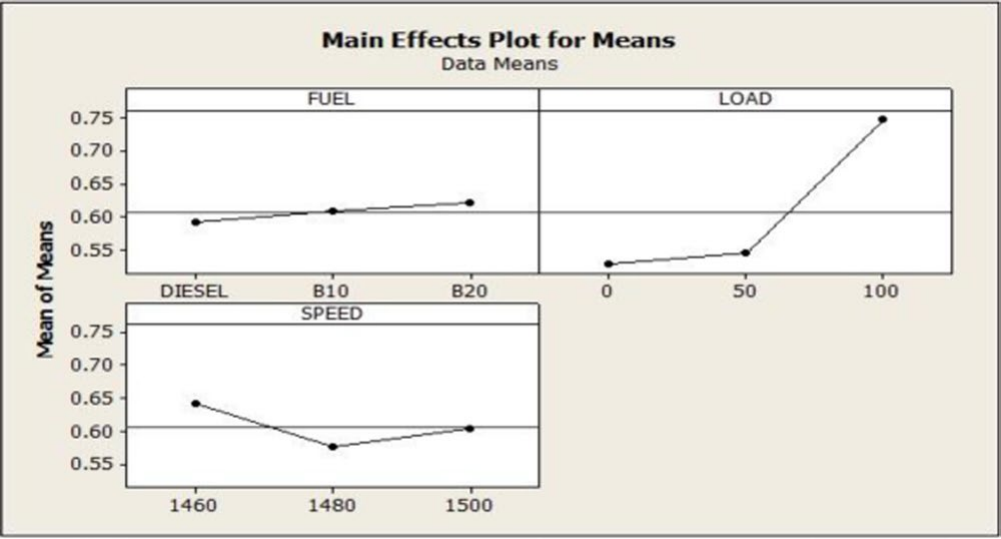
GRA ANOVA Analysis -The Plot Figure Indicates the GRA Optimum Parameters. (A3B3C1).

**Figure 10:**
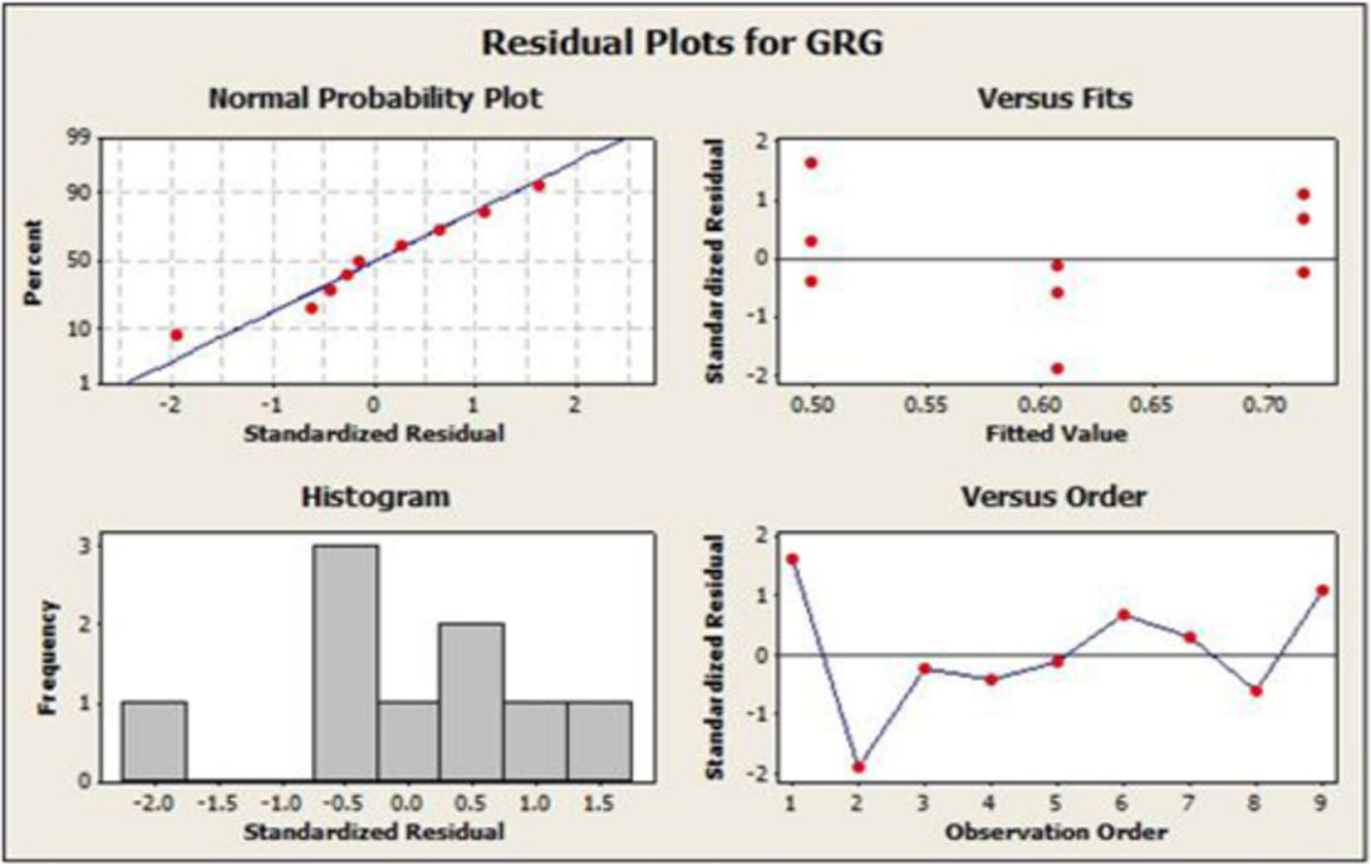
ANOVA Analysis Residual plots for GRG for Indicates the Variations of all the Nine Experiments.

Therefore the ANOVA of GRG analysis conclude that the second factor load poses the maximum mean square value of 0.0455 and hence it is identified as the highest significant factor that contributes and influences the performance and emission characteristics of a catalytic coated IC engine. The ANOVA mean effect plot and residual plots for GRG are shown in Figs. 9 and 10.

### Confirmation test

The final step of taguchi design technique is confirmation test. It is conducted through experimental work once again to validate the improvement of performance and to reduce emission characteristics in the coated piston and head type IC engine run by B20 blended diesel fuel. The identified optimum parameter responses BP, SFC, BTE, Torque, CO, HC, NO_X_ obtained through experimental and GRA are presented in Table 12. This shows the comparison of the experimental results using the initial OA, (A3B3C2-experiment no. 9) and optimal grey theory prediction design (A3B3C1) factor. Based on the confirmation test experimental result comparison, it clearly states that the performance and emission characteristics of a catalytic coated IC engine marginally improved through this study.

**Table 12:** Comparison of OA and GRG Optimum Parameters Results.

Condition description	Grey theory prediction design parameters	Based on OA Parameters
Level	A3B3C1	A3B3C2
BP in KW	0.9938	0.9940
SFC in Kg/kw-hr	0.321	0.3330
BTE in %	0.9572	1
TORQUE in N-m	0.9982	0.9926
CO in % Vol.	0.9872	1
HC in ppm	0.7052	0.7239
NOX in ppm	0.586	0.4489
GreyRelational Grade	0.8335	0.7846

Improvement in grey relational grade = 0.0489.

**Figure 11:**
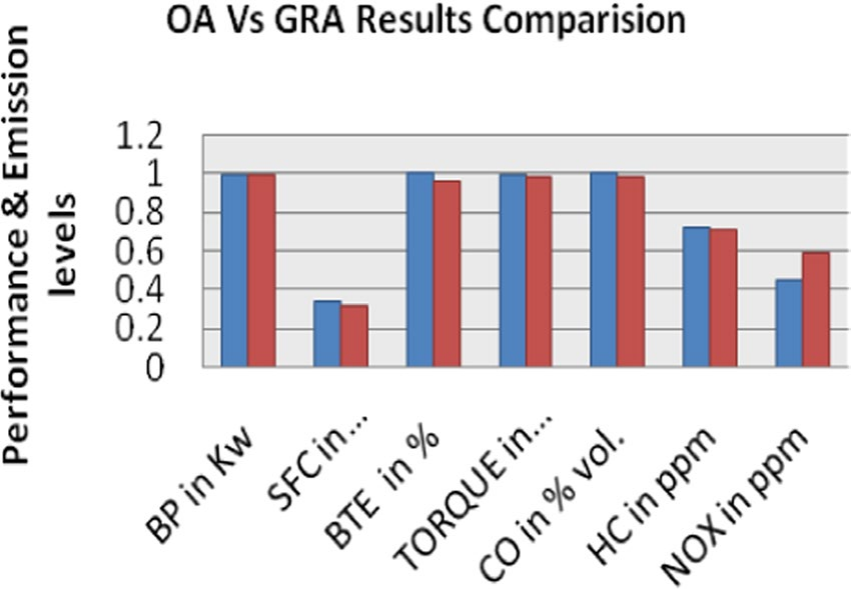
OA Vs GRA Engine Performance and Emission Comparison Results- It indicated the GRA prediction parameters experiment and showed better results in engine performance in SFC, Torque, CO, and HC emission.

The diesel engine combustion takes place in the combustion chamber in three stages, namely, ignition delay period, rapid combustion and controlled combustion. In that, ignition delay period implies higher influence in diesel engine combustion process. The delay period stage is dived into chemical delay and physical delay. The physical delay can be controlled by various factors such as fuel atomization, raise in pressure and temperature etc., The cylinder pressure and temperature rise depends upon speed and load of the engine. So the variation of speed changes from 1480 rpm to 1460 rpm at same load condition. The power output of engine BP and specific fuel consumption has slightly decreased and it is showed in the grey prediction design factor level (A3B3C1) results in Fig. 11. The thermal conductivity of copper chromium and zirconium catalytic cooper alloy material is higher, so the chemical reaction starts faster and then further accelerates to higher combustion rate by means of catalyst material and hence ignition delay period is reduced and complete combustion takes place in the second stage of rapid combustion. The combustion is further controlled up to the third stage when fuel droplets are injected till end. Due to the complete combustion, the CO and HC emission is reduced and NOx increases because of high heat flame temperature.

Based on the confirmation experiments, once again the verification experiments are conducted for comparing the results of both coated and un- coated engine. Based on the optimum parameter condition B20 blended diesel fuel, 100% load and 1460 rpm was maintained, and the new results are tabulated in Table 13. Based on the experimental test coating of piston along with bio diesel has resulting the reduction of CO and HC emission of a diesel engine because the coating and bio diesel leads to increased wall heat losses and lower wall temperature levels. Therefore, the objective of the research work has been fulfilled.

**Table 13:** Comparison of Uncoated- Diesel Engine (Base Engine) With Coated - bio diesel Engine.

Response Parameters	Un-Coated Engine run with diesel fuel	Coated Engine run with biodiesel fuel
BP in KW	3.3	3.3
BSFC in Kg/kw-hr	0.36	0.38
BTE in %	23.54	26.68
Load in kg	11.8	11.8
Speed in rpm	1460	1460
CO in % Vol.	1.01	0.01
HC in ppm	30	20
NOX in ppm	45	55

**Figure 12:**
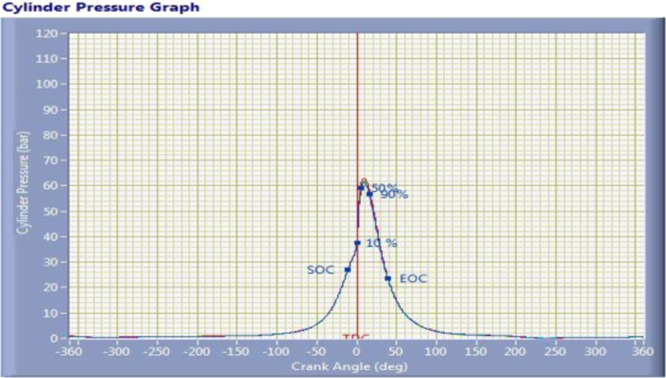
Cylinder Pressure Vs Crank Angle Plot –The image taken through “Engine LV Software” it indicates the maximum cylinder pressure and start and end of combustion with respect to crank angle (p-Ө diagram).

**Figure 13:**
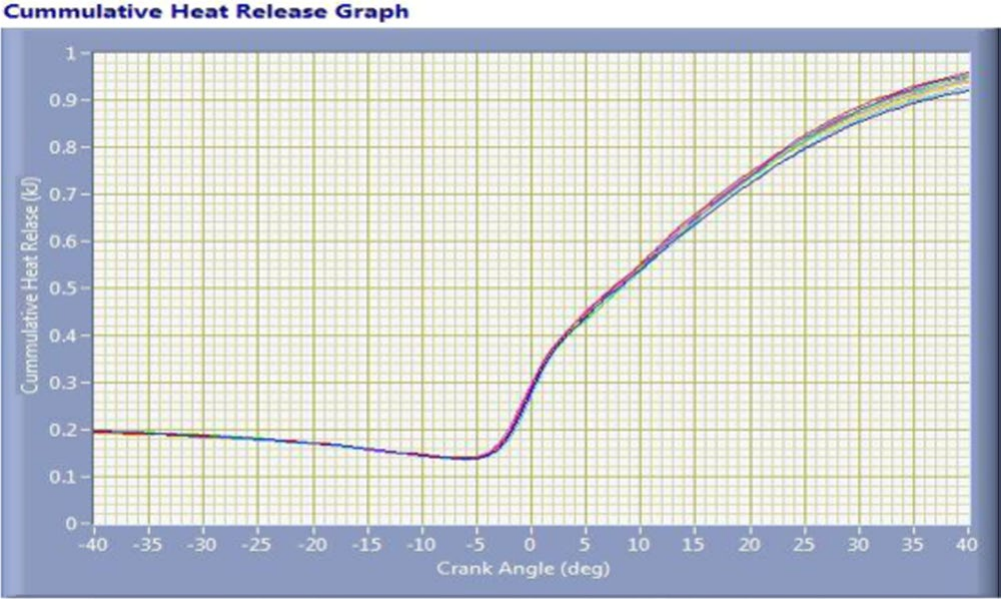
Crank angle Vs Cumulative Heat Release Plot- It Indicates heat release rate with respect to crank angle.

The cylinder pressure and heat release rate was varies depending upon crank angle at 1460 rpm speed and 11.8 kg load of a engine for coated engine as shown in Fig. 12. The maximum cylinder pressure developed in coated engine is 62 bar. The cumulative heat release for a coated engine is 0.95 KJ and it is shown in Fig. 13.

**Figure 14:**
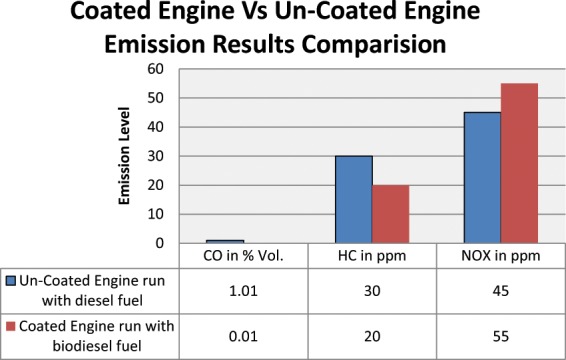
Coated and Un-Coated Engine Emission Results Comparison Plot. The plot Indicates CO and HC emission drastically reduced and Nox Increased due to high engine temperature operating conditions.

Ponnusamy *et al*.^7^ investigated the effect of copper coating copper gives improved performance and reduces emission, due to rapid flame propagation and catalytic activation of catalytic coating material present in the engine piston and combustion chamber which leads to reduce the CO and HC emission. Similarly in this investigasation the coated engine showed reduction of CO and HC emission marginally. It is shown in Fig. 14.

### Microstructure of coated piston

**Figure 15:**
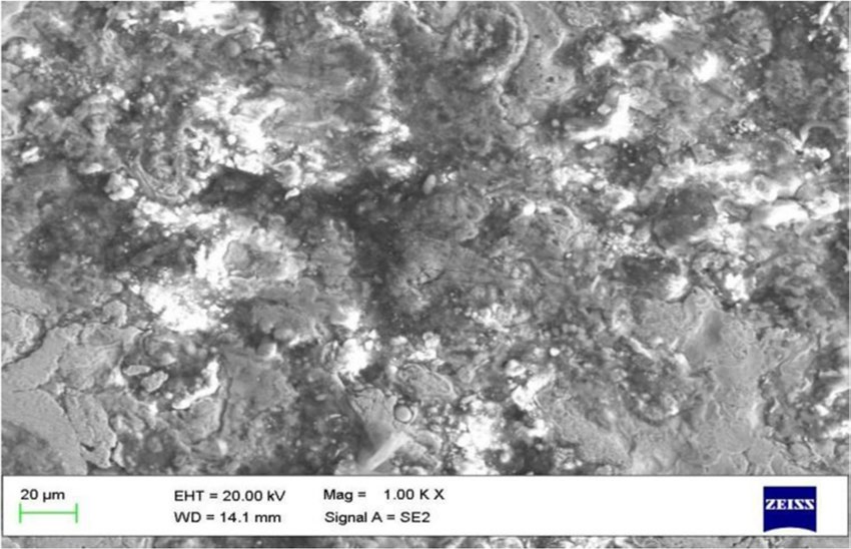
SEM image Indicates some cracks formed after combustion in the coated piston and no peeling of coating. Grain structure is varying from 10–20 μm, Magnification: 1.00 K X.

The scanning electron microscope (SEM) piston coating image is shown in Fig. 15. The above image is taken after running the experimental test. The grain structure is varying from 10–20 μm. In this image, some cracks are noticed on the top surface of piston coating area, and it is indicates the impact of thermo mechanical stresses developed during the working of engine. The presence of carbon particle on the black colored surface indicates the result of combustion and it is also noticed that bond coat and catalytic alloy coating material are attached firmly. There is no indication of peeling or melting of coating of top surface of the piston crown due to high engine temperature induced at the peak of combustion.

## Conclusions

Taguchi with GRA technique has been very efficiently used in the optimization of the performance and emission characteristics of a copper-chromium-zirconium alloy coated piston type IC engine. The three parameter that improve the performance and reduce the emission are identified as Fuel, Load and Speed. Cottonseed oil blended diesel is used as the fuel and the results are compared to that of the pure diesel. Experiments are conducted based on L9 orthogonal array and the engine parameters are optimized through the Taguchi-GRA experimental design method.

In the present investigation, Taguchi-GRA experimental design method is used to convert the multi response (seven responses) optimization problem into a single response problem. Based on the GRG result, B20 fuel, 100% load and 1460 rpm engine speed (A3B3C1) are identified as optimum parameter for copper chromium and zirconium catalytic coated piston type diesel engine.

The seven responses such as BP, BTh, Torque, SFC, CO HC and NOX have been determined for coated and uncoated engines. The brake thermal efficiency (BTh) of copper chromium and zirconium coated engine has marginally improved by 13.33% and CO, HC emissions have significantly decreased by 98.59% and 33.33.% respectively. This reveals that complete combustion takes place inside the combustion chamber due to piston coating on copper chromium and zirconium alloy material. And both engine producing the same brake power (BP) and not much variation in specific fuel consumption (SFC) and NOx emission increased due to engine high temperature.

Instead of diesel fuel operated engine, cotton seed oil - blended diesel (B20) fuel operated coated engine gives better results hence cotton seed oil-bio diesel can be used as an alternative fuel for controlling the emissions like CO, HC of a diesel engine.
